# Does macroscopic mass transfer affect sonochemical reaction rate in an ultrasonic bath?^☆^

**DOI:** 10.1016/j.ultsonch.2025.107361

**Published:** 2025-04-17

**Authors:** Takuya Yamamoto, Shinya Okino

**Affiliations:** a Department of Chemical Engineering, Graduate School of Engineering, Osaka Metropolitan University, 1-1, Gakuen-cho, Naka-ku, Sakai, Osaka 599-8531, Japan; b Department of Mechanical Engineering and Science, Graduate School of Engineering, Kyoto University, Kyoto daigaku-Katsura 4, Nishikyo-ku, Kyoto 615-8540, Japan; c Department of Mechanical Engineering, Tokyo Denki University, 5 Senju Asahi-cho, Adachi-ku, Tokyo 120-8551, Japan

**Keywords:** Ultrasonic degradation, Planer laser induced fluorescence (P-LIF), Mass transfer, Sonochemical reaction

## Abstract

In the present study, a planar laser induced fluorescence (P-LIF) measurement, reaction rate measurement, the sonochemical luminescence (SCL) observation, and the particle image velocimetry (PIV) measurement were conducted to clarify the effect of macroscopic mass transfer on sonochemical reaction rate in an ultrasonic bath. The concentration distribution was measured by the fluorescence intensity of Rhodamine 6G (Rh6G), which was illuminated by a CW-YAG laser sheet. The concentration of Rh6G decreases first in the high reaction zone measured by the SCL observation, and the resulting low-concentration zone expands to the low reaction zone through macroscopic convective mass transfer, which can be observed as solute plumes. Therefore, it is concluded that the mass transfer rate can slightly affect the chemical reaction rate due to the nonuniform concentration distribution in the early stage of sonochemical reaction. The reaction rate is slightly underestimated due to the spatial variation of the concentration in the early stage of ultrasonic degradation. The effect of macroscopic mass transfer on the sonochemical reaction rate was evaluated by first Damköhler number, which was calculated based on the flow velocity obtained by the PIV measurement and the reaction rate constant obtained by the decomposition experiment. Finally, it could be concluded that the first Damköhler number evaluates the effect of macroscopic mass transfer on the sonochemical reaction rate quantitatively and this dimensionless number can be applied to other ultrasonic bath with different condition.

## 1 Introduction

When high-intense ultrasound is irradiated into an aqueous solution, acoustic cavitation occurs. This acoustic cavitation causes many phenomena. The cavitation bubbles are formed during ultrasonic oscillations, and the bubble is oscillated nonlinearly because the phase shift occurs between the bubble and ultrasonic oscillations. Due to this phase shift, the bubble is largely compressed, and at this moment, the bubble inner temperature and pressure instantly becomes higher than 5000 K and 100 atm., respectively [[1], [2], [3]]. Therefore, the chemical species in the bubble are thermally decomposed, causing the generation of radicals, which react with chemicals in an aqueous solution. In addition to this chemical effect, the dynamic behavior of cavitation bubbles causes many physical effects. For example, a microjet is formed under the asymmetric conditions where the bubble is largely compressed due to nonlinear oscillations [[4], [5], [6], [7], [8], [9]], a bubble cluster is formed [[10], [11], [12], [13]], and shockwaves are emitted when cavitation bubbles are collapsed [14]. These chemical and physical phenomena occur simultaneously in an aqueous solution, and these phenomena have been used for many applications such as ultrasonic degradation [15,16], atomization [17,18], emulsification [[19], [20], [21], [22]], fabrication of organic and inorganic materials and etc. [23,24]. In this study, we focus on the interaction between chemical and physical effects during ultrasonic irradiation into an aqueous solution.

The chemical effects during ultrasonic irradiation have been widely investigated especially for ultrasonic degradation and environmental use. To evaluate chemical effects in an ultrasonic bath, many methods have been used. The most common method to quantify the chemical effects is sonochemi luminescence (SCL) observation [25]. The ultrasonic bath is filled with a luminol aqueous solution, and the chemiluminescence of luminol solution is recorded by a detector or camera. This SCL observation can measure the reaction zone in the ultrasonic bath. In addition to this method, other methods have been also used to quantify the chemical reaction rate in a whole ultrasonic bath such as Fricke dosimetry, KI dosimetry, Weissler reaction, decomposition method, and etc. [[26], [27], [28], [29], [30], [31]]. Although the chemical reaction rate has been evaluated through the above dosimetry and methods, the chemical reaction rate is also affected by the spatial distribution of chemicals in a bath [32]. For example, the chemical species are required to be transported into the reaction zone in the decomposition method. Therefore, the chemical reaction rate obtained through the above quantitative methods is an apparent rate [[33], [34], [35]], and one needs to consider the effect of macroscopic mass transfer induced by ultrasonic irradiation (e.g., acoustic streaming and flow induced by bubbles motion) on the chemical reaction. The effect of macroscopic mass transfer on the chemical reaction rate is difficult to be evaluated in the ultrasonic bath. To investigate the effect of macroscopic mass transfer on the chemical reaction rate, one can consider the addition of an external flow in an ultrasonic bath [36]. However, the external flow in the ultrasonic bath also changes not only the macroscopic mass transfer but the reaction zone and cavitation activity [[37], [38], [39]]. Another way to enhance the macroscopic mass transfer is that the ultrasonic power and ultrasonic bath are enlarged so that they have the same ratio [40]. However, this method can change the ultrasonic and reaction fields. For these reasons, the influence of macroscopic mass transfer on the reaction rate has not been clarified yet.

**Fig. 1:**
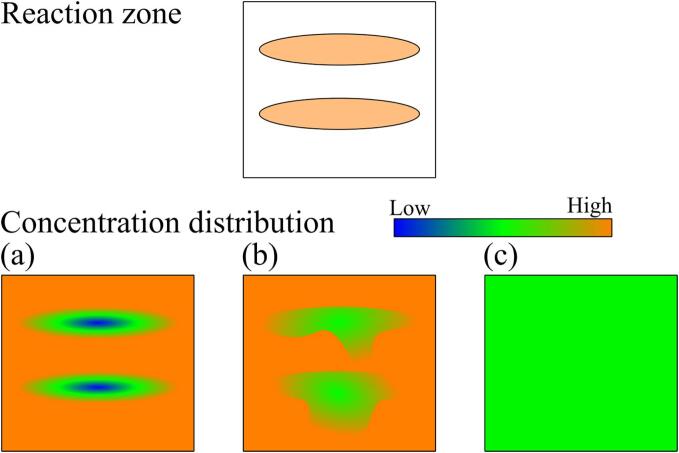
Possible scenario of macroscopic concentration distribution: (a) high reaction rate and low mass transfer rate, (b) middle reaction rate and middle mass transfer rate, and (c) low reaction rate and high mass transfer rate.

To understand this phenomenon, we measured the spatial concentration distribution of chemical species in an ultrasonic bath through a planar laser induced fluorescence (P-LIF) measurement for the ultrasonic degradation in this study. The possible scenario of macroscopic concentration distribution in an ultrasonic bath is shown in Fig. 1. We assumed the situation where an ultrasound is irradiated from the bottom and a standing wave is formed. The chemical reaction rate is high in the horizontal band zone marked in orange color in Fig. 1 (a). The concentration distribution of chemical species is determined by the balance between the chemical reaction rate and mass transfer rate. The concentration of chemical species becomes low only in the band-shaped high reaction zone when the reaction rate is high, whereas the concentration is almost uniform when the chemical reaction rate is very low as shown in Fig. 1 (c). The concentration plume structure can be seen when the reaction rate and mass transfer rate are comparable as shown in Fig. 1 (b). By quantifying the spatial concentration distribution of chemical species, we discussed the possibility whether the macroscopic mass transfer affects the apparent chemical reaction rate or not.

## 2 Experimental procedures

**Fig. 2:**
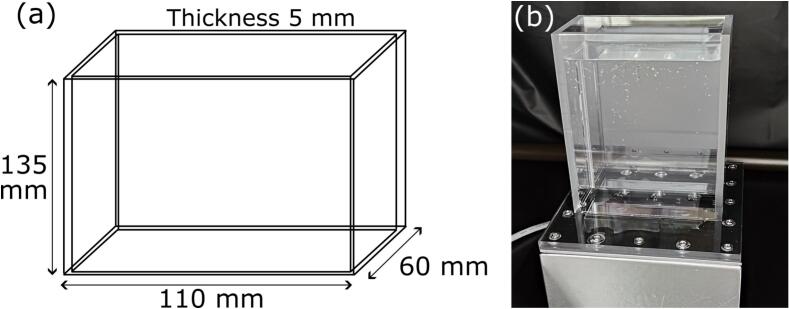
Ultrasonic vessel used in this study: (a) dimension of the vessel and (b) photograph of the vessel.

To evaluate the macroscopic distribution of dye concentration, we conducted the following five experiments: (i) calorimetry method, (ii) measurement of degradation rate of dye, (iii) measurement of sonochemical luminescence (SCL), (iv) P-LIF measurement, and (v) particle image velocimetry (PIV) measurement. In experiments (i) and (ii), the interaction between the reaction rate and the ultrasonic power was evaluated. In experiment (iii), the reaction zone was visualized. In experiment (iv), the macroscopic distribution of dye concentration was measured. Many researchers have used Rhodamine B, Methyl Orange or typical dye to evaluate the ultrasonic degradation rate [26,31,41]. However, in this study, Rhodamine 6G (Rh6G) was used because it is more suitable for the P-LIF experiment. Rh6G has fluorescence properties, and the absorbance wavelength is very close to green laser with the wavelength of 532 nm. Besides, the temperature dependency of fluorescence property of Rh6G is smaller than that of Rhodamine B [42]. The fluorescence disappears due to chemical reactions with radicals generated from acoustic cavitation as explained later in the result section. Besides, Rh6G has low degree of photobleaching [43,44]. To irradiate the laser sheet throughout the vessel, the vessel wall except for the bottom part of the transducer is made of acrylic. The dimensions and photograph of ultrasonic vessel are depicted in Fig. 2. In this vessel, the inner length was 100 mm in the width, 50 mm in the depth, and 135 mm in the height. The acrylic cover was fixed by bolts to the periphery of oscillation plate, which is connected to the transducer with a rubber plate in between. The rubber plate was cut out only on the irradiation surface of ultrasound. In this study, the 600 mL aqueous solution was used, and the solution height was 120 mm. The ultrasound was irradiated from the bottom transducer, and the ultrasonic frequency was 153 kHz because the sonochemical efficiency is large at approximately 100–300 kHz [26,45]. The electric power was varied from 20 to 200 W. In experiment (v), the fluid flow velocity of acoustic streaming was measured.

### 2.1 Calorimetry method

To evaluate the conversion efficiency of electric power into the ultrasonic power, we first measured the heating rate of water in the ultrasonic vessel. The whole part of ultrasonic vessel was covered by a heat insulator made of glass fibers, and the temperature in water was measured by a K-type thermocouple (Okazaki Manufacturing company, AEROPAK). In this method, the ultrasonic power, *P*_US_ [W] was calculated as

(1) $$P_{\mathrm{US}}=MC_{p}\frac{\mathrm{dT}}{\mathrm{dt}}$$

where *M* is the mass of water [kg], *C*_p_ is the heat capacity [J/(kgK)], *T* is the temperature [K], and *t* is time [s]. By measuring the time variation of solution temperature, the differentiation of *T* with respect to *t* was calculated. The experiment was repeated three times, and the standard deviation and the averaged values of *P*_us_ were calculated.

### 2.2 Degradation rate of Rhodamine 6G

In the same vessel, the degradation rate of Rh6G was measured to evaluate the reaction rate and efficiency. We took 3 mL aqueous solution at regular intervals, and the time variation of Rh6G concentration was measured by the ultraviolet–visible absorption spectroscopy (UV-2600, Shimazu). The sample was taken every 2 min. for the high-power condition, and longer interval for the low-power condition because the concentration change was smaller for the low-power condition. After the absorption measurement, the used sample was returned into the vessel. This measurement was continued until five concentrations were obtained at different time including the initial one. The solution concentration was calculated using the Lambert-Beer law described as

(2) A=aC

where *A* is the absorption [-], *a* is a constant [L/g], and *C* is the solution mass concentration [g/L]. Initially, the constant, *a* was calculated by changing the solution concentration. Then, the absorption of degraded Rh6G aqueous solution was converted into the solution concentration.

The sonochemical reaction is pseudo-first order reaction [46]. So, to calculate the reaction rate constant, *k* [/s], we fit the experimental data to the exponential function as:

(3) $$C/C_{0}=\exp\left(-kt\right)$$

In studies of sonochemical reaction, the sonochemical efficiency and the reaction rate have been widely used to evaluate the reaction rate. Meanwhile, in the study of sonochemical degradation, the reaction efficiency and reaction rate are time-dependent and decrease with time. Hence, in this study, the reaction rate in the ultrasonic bath, *G* [mol/s], and the sonochemical efficiency, *SE* [mol/J], were evaluated at the initial time of ultrasonic irradiation, that are calculated as

(4) $$G=k{C^{\prime }|}_{t=0}V$$

(5) $$\mathrm{SE}=\frac{k^{'}}{P_{\mathrm{US}}}$$

where *V* is the solution volume [L], *C*’ is the molar concentration [mol/L], which is calculated as

(6) $$CM_{w}=C^{'}$$

*M*_w_ is the molar weight [g/mol].

To control the initial concentration of dissolved gases, air was injected into the solution for 15 min. before the ultrasonic irradiation. The initial concentration of Rh6G was 100 μg/L, which is 10 times larger than that used in the P-LIF experiment, because the absorption spectrometer used in this study cannot accurately evaluate the absorption of 10 μg/L Rh6G aqueous solution. The experiment was repeated three times, and the averaged value and the standard deviation were calculated.

### 2.3 Sonochemical luminescence (SCL) observation

In addition to the reaction rate, the reaction zone was measured by sonochemical luminescence (SCL). In this experiment, the chemiluminescence of luminol aqueous solution was used to visualize the reaction zone. The concentration of luminol was 0.01 wt%, and Na_2_CO_3_ was also added to keep the aqueous solution alkaline. The concentration of Na_2_CO_3_ was 0.5 wt%. In the same way as the degradation experiment, we filled the vessel with this luminol solution, the volume of which was 600 mL. The SCL was recorded by electron multiplying (EM-) CCD camera (Andor, iXon EMCCD DU-888U3-CS0-EXF). The frame rate and exposure time were 5 fps and 0.1 s. We took photographs 10 s after the ultrasonic power was turned on because the ultrasonic field was unstable just after the power supply. The electron multiplying gain was not used. Hence, the EM-CCD camera was used as a normal CCD camera.

### 2.4 Planer laser induced fluorescence (P-LIF) measurement

**Fig. 3:**
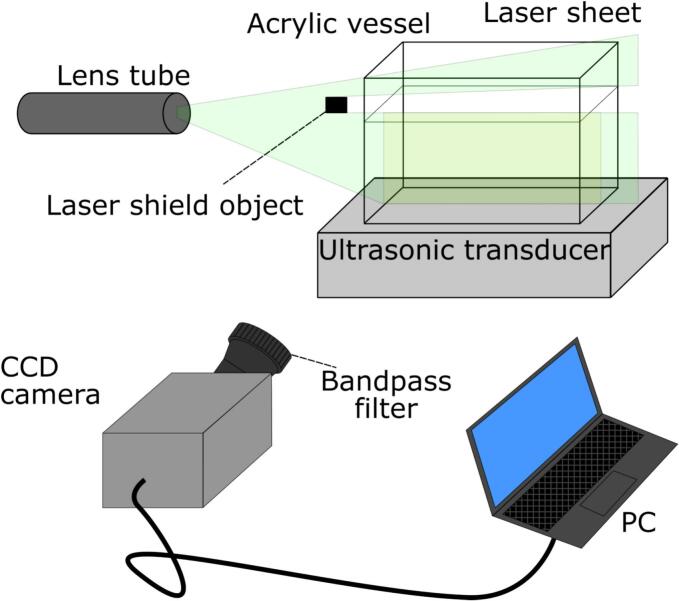
Schematic drawing of planer laser induced fluorescence (P-LIF) equipment.

The experimental apparatus of P-LIF measurement is shown in Fig. 3. In this measurement, the initial concentration of Rh6G was 10 μg/L, and the solution volume was 600 mL. The Nd:YAG continuous wave laser (Kanomax, CW532-3WM) was used for the sheet light source. The wavelength of this laser was 532 nm, which corresponds to the absorbance peak wavelength of Rh6B solution, that was approximately 525–530 nm. The spot laser was converted into the sheet laser through two cylindrical lenses. A part of laser sheet was masked by a laser shield object near the free surface because the dynamic motion of free surface creates the irregular reflection at the free surface, that changes the fluorescence intensity largely with time. This irregular reflection largely deteriorates the measurement accuracy. To prevent this reflection, the 10 mm height of solution near the free surface was shaded. The fluorescence intensity was measured by an EM-CCD camera (Andor, iXon EMCCD DU-888U3-CS0-EXF). In this case, the electron multiplying gain was not used in the same way as it was not used in the SCL measurement. In front of the camera lens, a bandpass filter (Edmund, Bandpass filter, #67–047) was attached. The bandwidth and the center wavelength are 40 nm and 562 nm, respectively. This filter cuts off the light from the laser sheet and passes the fluorescence light. The exposure time was 0.1 s, and the frame rate was 1.0 fps. The color depth of obtained picture was 16 bit. The spatial resolution of this camera was 1024 × 1024, and the pixel binning of 4 × 4 was used. That is, the spatial resolution obtained from this measurement was 256 × 256. The ultrasonic degradation is pseudo-first order reaction, the chemical reaction rate of which is the largest in the early stage of ultrasonic irradiation. Therefore, the time variation of fluorescence intensity was recorded for a short period, namely 120 s.

Before the measurement of concentration distribution, the fluorescence intensity was recorded for three different solution concentration: 5.0, 7.5, and 10 μg/L as the reference data. The fluorescence intensity temporarily varied slightly due to the stability of the laser light. Therefore, the time-averaged fluorescence intensity was used as the reference data. The number of time averaging was 10, and the time interval of each picture was 5 s. The relationship between the obtained distribution of fluorescence intensity and the concentration distribution of Rh6B for each pixel was approximated by the following equation.

(7) C=aI+b

where *C* is the concentration of Rh6G, *I* is the light intensity, and *a*, *b* are the constant to be determined for each pixel by the reference data. By using this relationship, the fluorescence intensity was finally converted into the solution concentration.

### 2.5 Particle image velocimetry (PIV) measurement

The experimental method for the PIV measurement was similar to that used in our previous study [47]. In this method, the fluid flow velocity was measured by small particles motion, which was recorded by a high-speed camera (Photron, FASTCAM Mini AX100). Fluorescent particles (Kanomax, EB-FLUOSTAR) with the diameter of 15 μm were dispersed into water, and a laser sheet was used to illuminate the fluorescent particles by CW-YAG laser (Kanomax, CW532-3WM). The laser sheet was irradiated to the center of the ultrasonic bath, and the wavelength of laser was 532 nm. The irradiation location of laser sheet was the same as that of P-LIF method as shown in Fig. 3. The fluorescent particles were excited by this laser, and the fluorescent light with the peak wavelength of 580 nm was emitted by the particles. A high-pass filter was used to eliminate the reflected light from the vessel and cavitation bubbles. The cut-off wavelength of this filter was 570 nm. Therefore, only the particles motion can be recorded by the high-speed camera. The framerate of highspeed camera was 250 fps, and the recording time duration was 20 s. The recorded particles motion was converted into the fluid flow velocity by a digital image correlation method (OpenPIV, Python opensource package).

## 3 Results and discussion

### 3.1 Characterization of ultrasonic bath used in this study

**Fig. 4:**
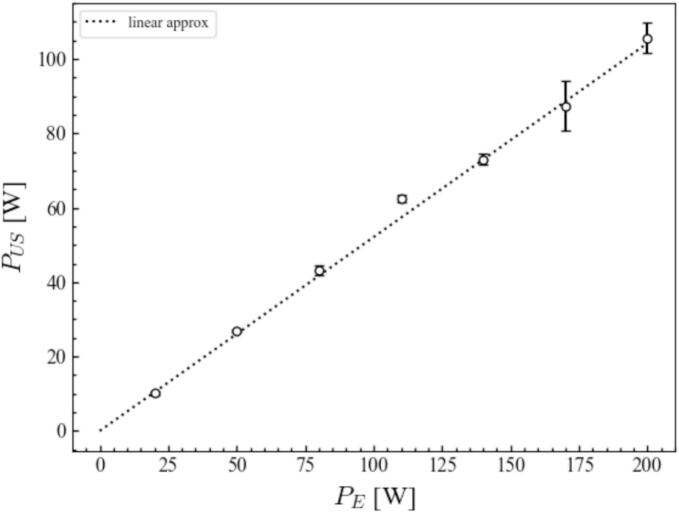
Relationship between the electric power, *P*_E_ and the ultrasonic power measured by the calorimetry method, *P*_US_. The relationship between *P*_E_ and *P*_US_ is *P*_US_ = 0.521 *P*_E_.

We first evaluated the conversion ratio of ultrasonic power. Fig. 4 shows the relationship between the electric power and the ultrasonic power measured by the calorimetry method. This relationship is almost linear. Therefore, in the following results, we used the ultrasonic power calculated by this approximate line. The conversion rate, *P_US_/P_E_*, is approximately 52.1 % in this experimental equipment. As described later, the experiments were mainly conducted at the electric power of 50 and 110 W with the irradiation time being up to 120 s. At the electric power of 110 W, where the temperature increases the most, the temperature increase is approximately 3 K in 120 s for this calorimetry experiment. In other experiment such as the SCL observation and the PIV measurement, the temperature increase becomes smaller than this value because the heat insulator was not used. It can be said that the effect of temperature increase on the reaction rate and fluid flow velocity is negligibly small. In the following sections, we will mainly explain the phenomena in terms of electric power for the sake of clarity. It is to be noted that in order to elucidate the general phenomena during ultrasonic irradiation, we should discuss them in terms of ultrasonic power.

**Fig. 5:**
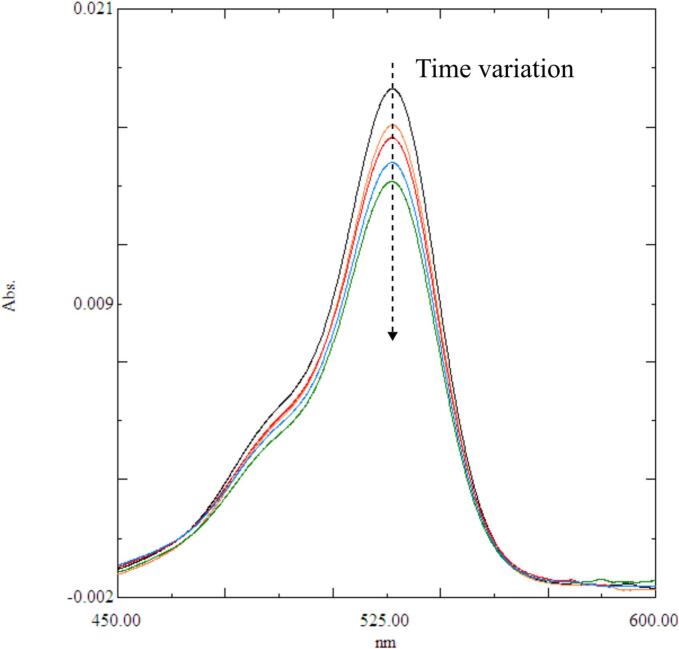
Time variation of absorption spectrum of Rh6G during ultrasonic degradation at the electric power of 20 W. Each spectrum was measured at every 5 min, and the color means the different sampling time.

The chemical reaction rate was evaluated by the degradation of Rh6G. As an example, the time variation of absorption spectrum for the Rh6G aqueous solution is shown in Fig. 5. In this result, the electric power was 20 W, and the absorption spectrum was measured at every 5 min. The ultrasonic irradiation decomposes the Rh6G due to generated radicals, and the intensity of absorption spectrum decreases with time. The peak absorption wavelength of Rh6G is approximately 525–530 nm, the peak intensity was used to quantify the solution concentration.

**Fig. 6:**
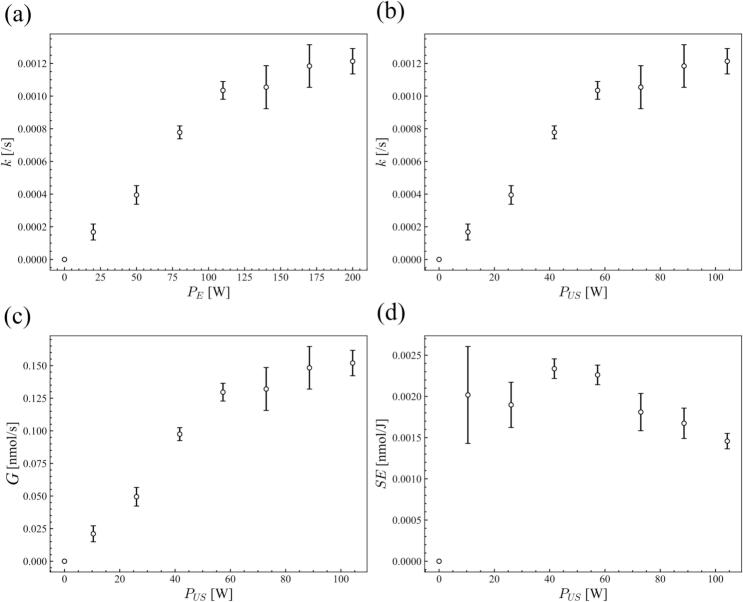
Characteristics related to chemical reaction for the Rh6G degradation at different ultrasonic powers: (a) relationship between the electric power and the reaction rate constant, relationships between the ultrasonic power and (b) the reaction rate constant, (c) the reaction rate at *t* = 0, and (d) the sonochemical efficiency at *t* = 0.

Fig. 6 shows the characteristics related to chemical reaction rate for Rh6G degradation at different ultrasonic powers. The chemical reaction rate and the SE were calculated by Eqs. (3), (4), (5), (6). The chemical reaction rate increases almost linearly with the ultrasonic or electric power when the power is low. However, the reaction rate becomes unchanged over *P*_E_ = 110 W. This phenomenon is well-known as initial trend of quenching [45,[48], [49], [50]]. As shown later, the pattern of reaction zone is changed at this power. In accordance with the reaction rate constant and reaction rate, the sonochemical efficiency decreases for the higher-power condition.

**Fig. 7:**
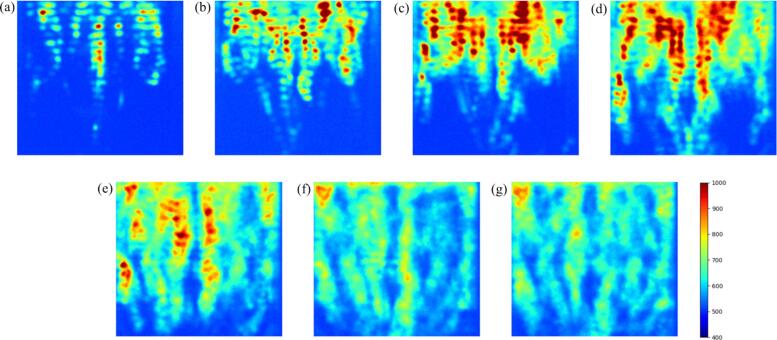
Reaction zone obtained by the SCL measurement with different electric powers: (a) 20 W, (b) 50 W, (c) 80 W, (d) 110 W, (e) 140 W, (f) 170 W, and (g) 200 W.

Fig. 7 shows the reaction zones obtained by the SCL measurement with different powers. The distribution of SCL intensity does not vary over time, although this intensity fluctuates slightly. The reaction zone exhibits a striped pattern in the horizontal direction at low power conditions. In these cases, the superposed ultrasound creates the standing wave. The wavelength of ultrasound can be calculated as

(8) $$\lambda =\frac{c}{f}$$

where λ is the wavelength of ultrasound, *c* is the speed of sound, *f* is the frequency. In this experimental condition, the wavelength is approximately 9.8 mm. The number of ultrasonic waves is almost 12 because the depth of Rh6G solution is 120 mm. When a clear standing wave is formed, the number of antinodes is twice of the number of ultrasound waves, i.e. 24 in this experimental condition. As seen from Fig. 7, the number of high-reaction stripe is almost 22–23 although the reaction zone in the bottom part is difficult to be distinguished. This is another evidence of standing wave formation. When a standing wave is formed, the oscillation amplitude of acoustic cavitation is large and the ultrasonic power is efficiently converted into the cavitation activity resulting in the higher rate of sonochemical and degradation reactions. At the higher power conditions, this clear high-reaction stripe becomes more unclear with increasing the power. This phenomenon is caused by deterioration of ultrasound superposition, which decreases the pressure amplitude of ultrasound and the oscillation amplitude of cavitation bubble becomes small, causing small sonochemical reaction rate [51]. According to this deterioration of superposition, the chemical reaction rate becomes almost constant, and the chemical quenching starts at this power as shown in Fig. 6.

In addition to the striped pattern, the location of macroscopic reaction zone is also changed with the power. At the small power, the reaction zone is confined to the upper part of vessel, and the reaction zone expands toward the bottom with increasing the power. This kind of pattern can be observed in other studies [51,52]. The higher reaction rate near the free surface was explained by the formation of standing wave near the free surface [51]. The ultrasound emitted from the bottom transducer propagates to the upper free surface, and the ultrasound is reflected at the free surface. Near the free surface, the superposition of ultrasound between reflected and incident waves is good causing the higher reaction rate [3,51].

As seen from the results of sonochemical reaction rate shown in Fig. 6 (a), the reaction rate becomes unchanged, and the SE decreases when the electric power becomes larger than 140 W. In addition to this phenomenon, the fluctuation amplitude of free surface increases with increase in the electric power leading to less accuracy of concentration measurement through the P-LIF measurement because the reflected laser sheet on the free surface fluctuates the light intensity. Furthermore, when the electric power is below 110 W, the reaction zone is localized, and the reaction zone is limited only in the upper zone of vessel as shown in Fig. 7. In this case, the influence of chemical reaction on the whole concentration distribution of Rh6G is more distinguishable because we can easily compare the concentration distribution in the high reaction zone of upper part with that in the low reaction zone of lower part. Therefore, the P-LIF measurement was conducted below 110 W in the subsequent section.

### 3.2 Planer laser induced fluorescence (P-LIF) measurement

Before the measurement of concentration in the ultrasonic vessel through the P-LIF measurement, we evaluated the degradation of Rh6G due to laser irradiation, which is called photobleaching. The experimental results are summarized in Appendix A. The degradation of Rh6G due to laser irradiation in our experimental apparatus is negligibly small. Therefore, the distribution of fluorescence intensity can be accurately converted to the concentration distribution of Rh6G. In addition, the reference fluorescence intensity was first measured with different concentration of Rh6G. The reference distribution of fluorescence intensity is shown in Appendix B. By using this reference data, the solution concentration was calculated.

**Fig. 8:**
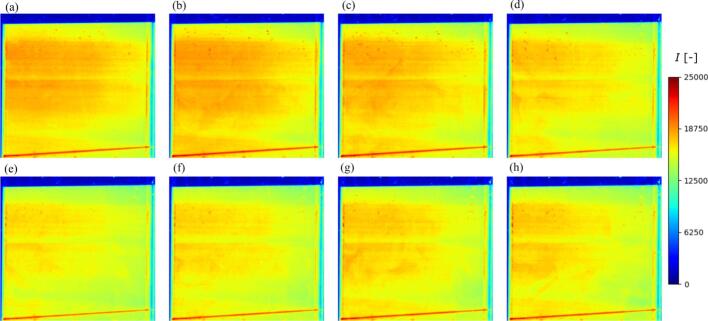
Time variation of distribution of fluorescence intensity at 50 W. The time increment for each picture was 5 s, and the irradiation time of ultrasound was (a) 5, (b) 10, (c) 15, (d) 20, (e) 25, (f) 30, (g) 35, and (h) 40 s. The initial concentration of Rh6G is 10 μg/L.

Fig. 8 shows the time variation of distribution of fluorescence intensity at the electric power of 50 W. The fluorescence intensity decreases with time, and many circular spots with a high fluorescence intensity are created especially in the upper part. These circular spots are caused by the bubbles attached to the front acrylic wall. In the measurement of concentration distribution in an ultrasonic vessel, these bubbles attachment is unavoidable, although these spots are measurement error. In addition, some low intensity stripped zones are formed in the horizontal direction. These zones are caused by the light blocking at bubbles attaching to the side wall. In the P-LIF measurement for an ultrasonic bath, these two types of measurement errors are unavoidably introduced. The bubble generation locations and the generation rate cannot be controlled. Therefore, we conducted several P-LIF measurements, and we selected the results with smaller measurement errors.

**Fig. 9:**
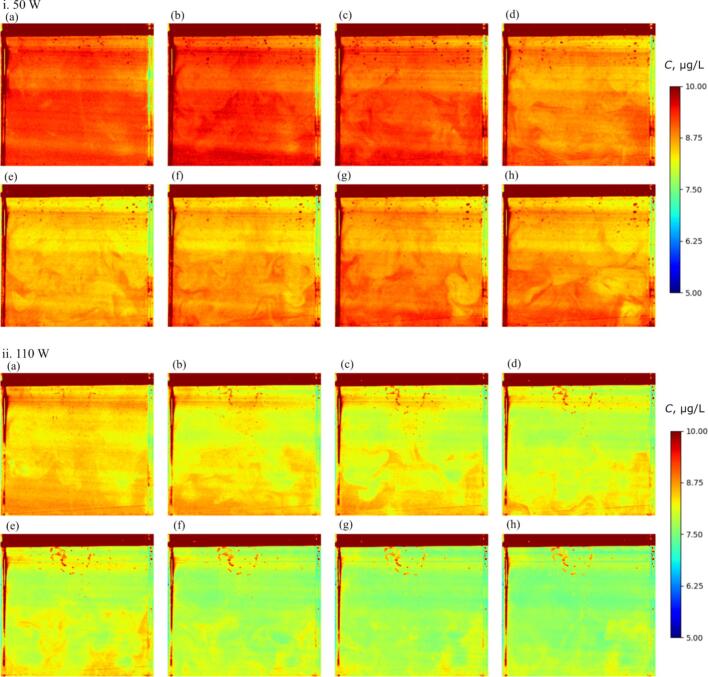
Time variation of concentration distribution of Rh6G at different electric powers: i. 50 W, and ii. 110 W. The time increment for each picture was 5 s, and the irradiation time of ultrasound was (a) 5, (b) 10, (c) 15, (d) 20, (e) 25, (f) 30, (g) 35, and (h) 40 s. The concentration distribution at 50 W was calculated from the fluorescence intensity distribution shown in Fig. 9.

Fig. 9 shows the time variation of concentration distribution of Rh6G at different electric powers (50 and 110 W). In this figure, the time increment of each picture is 5 s. In both electric powers, the Rh6G is decomposed into other chemical species causing the decrease in the fluorescence intensity. Also, the time variation of average concentration in this figure roughly corresponds to the reaction rate obtained in Fig. 6. Besides, the decompose rate is higher in the case of 110 W. Even though some high-concentration spots and the horizontal striped pattern are seen due to the unavoidable measurement errors in this figure, the concentration distribution of Rh6G could be measured. In both electric powers, the decompose rate of Rh6G is higher, and the concentration becomes lower in the upper zone where the rate of sonochemical reaction is higher as shown in Fig. 7. The lower concentration zone spreads toward the bottom part of vessel through solute plumes. This result indicates that the convective mass transfer plays a crucial role for the whole mass transfer and concentration distribution. The difference between the maximum and minimum concentrations is almost 1.0 – 2.0 μg/L at most.

**Fig. 10:**
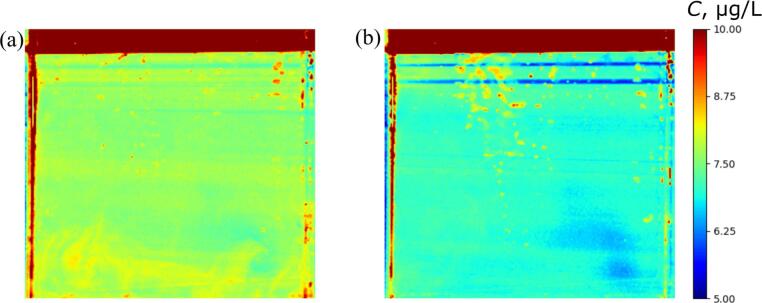
Concentration distribution of Rh6G for the electric power of (a) 50 and (b) 110 W at 120 s.

Fig. 10 shows the concentration distribution of Rh6G for the electric power of 50 and 110 W at 120 s. As the irradiation time increases, the bubbles nucleate at the acrylic vessel walls, causing many spurious high concentration spots and horizontal stripped pattern. Although the accuracy of concentration distribution is getting worse with longer irradiation time, the spatial concentration difference becomes lower. With increase of the ultrasonic irradiation time, the reaction rate decreases because this degradation reaction is pseudo-first order reaction, and the convective mass transfer rate becomes larger than the reaction rate.

### 3.3 Particle image velocimetry (PIV) measurement

**Fig. 11:**
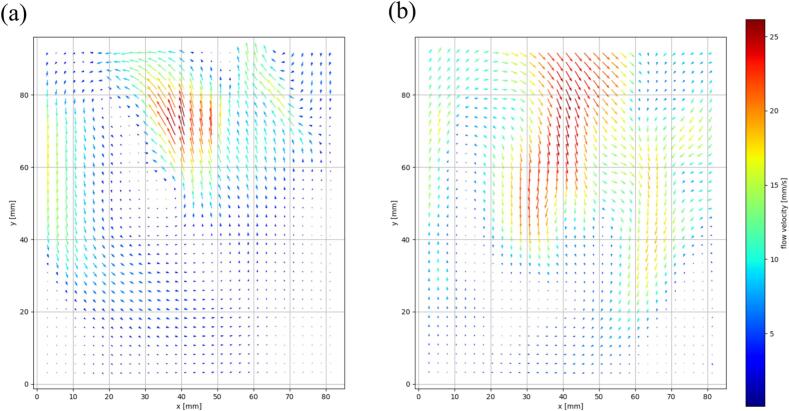
Time-averaged flow velocity vectors obtained by the PIV measurement at the electric power of (a) 50 and (b) 110 W.

To evaluate the convective mass transfer, the fluid flow velocity was measured. The ultrasonic power in this experiment was 50 and 110 W. Fig. 11 shows the time-averaged flow velocity vectors obtained by the PIV measurement. The magnitude of the maximum flow velocity is almost the same for both powers. On the other hand, the flow velocity is large in a wide zone for 110 W, and the flow direction in the middle upper part is changed by the ultrasonic power. Based on this flow distribution and flow velocity, we will discuss the effect of macroscopic mass transfer on the sonochemical reaction rate in the following section.

### 3.4 Discussion about the effect of macroscopic mass transfer on the reaction rate

As studied in Koda *et al*. [26] and Asakura and Yasuda [45], the reaction efficiency is the maximum near the frequency of 200 kHz, which is close to 153 kHz used in this study. Also, the high-reaction rate condition was selected as explained in Fig. 6, Fig. 7. Therefore, the almost highest reaction rate condition was selected in an ultrasonic degradation without additional chemicals [[53], [54], [55]] and dissolved gas control [56,57]. As shown in Fig. 9, Fig. 10, the concentration distribution of Rh6G is not uniform, and the solute plumes was observed especially in the early stage of ultrasonic degradation. In the scenario as shown in Fig. 1, the ultrasonic degradation has the middle reaction rate and middle mass transfer rate. Therefore, the ultrasonic degradation was assumed to be under the condition where the mass transfer rate is comparable to the sonochemical reaction rate for an ultrasonic bath.

Next, the influence of spatial concentration variation on the chemical reaction rate is discussed. The concentration varies spatially by approximately 20 % at most. We discuss whether this variation can affect the reaction rate or not. In the pseudo-first order reaction, the time variation of concentration is described as Eq. (3). Although the reaction rate was measured in Fig. 6, this reaction rate is apparent because the dye in the vessel is not uniformly distributed. Here, we defined the actual reaction rate constant, *k*_act_ and the apparent reaction rate constant, *k*_app_. The apparent reaction rate, *k*_app_ is underestimated because the higher concentration zone does not reach to the reaction zone. As discussed above, we considered the most non-uniform condition that the concentration at the reaction zone is low by 20 %. In this case, the actual reaction constant is roughly described as:

(9) $$k_{\mathrm{act}}=-\frac{\ln\left(0.8\frac{C}{C_{0}}\right)}{t}$$

which leads to

(10) $$k_{\mathrm{act}}=-\frac{\ln\left(0.8\right)}{t}-\frac{\ln\left(\frac{C}{C_{0}}\right)}{t}=\frac{0.2231}{t}+k_{\mathrm{app}}>k_{\mathrm{app}}$$

From this relation, we can state that the reaction rate constant can be underestimated especially in the early stage of ultrasonic irradiation. Meanwhile, as seen from Fig. 9, the concentration spatial difference becomes large after 10 s. In addition, the concentration spatial difference becomes small with time for a long irradiation time as shown in Fig. 10. From this discussion, the influence of macroscopic mass transfer on the reaction rate is small when the sampling rate of solution for the concentration measurement is set to be sufficiently high or sufficiently long, although it can affect in the early stage of ultrasonic degradation.

To create a general way to evaluate the effect of macroscopic mass transfer on a sonochemical reaction rate under different conditions, we introduced dimensionless parameters. The macroscopic phenomena can be described by the following reaction–diffusion equation.

(11) $$\frac{\partial C}{\partial t}+u\cdot \nabla C=D\nabla^{2}C-kC$$

where *D* is the diffusion coefficient of Rhodamine 6G in the solution. This equation is non-dimensioned by introducing the following dimensionless numbers:

(12) $$x^{*}=\frac{x}{L},C^{*}=\frac{C}{C_{0}},u^{*}=\frac{u}{U_{0}},t^{*}=\frac{t}{L/U_{0}}$$

where *L* is the characteristic length, *U*_0_ is the characteristic fluid velocity, the superscript * indicates the dimensionless. The reason why this non-dimensionalization was used is described in Appendix C. By substituting Eq. (12) into Eq. (11), the following dimensionless reaction–diffusion equation can be obtained.

(13) $$\frac{\partial C^{*}}{\partial t^{*}}+u^{*}\cdot \nabla C^{*}=\frac{D}{LU_{0}}\nabla^{2}C^{*}-\frac{\mathrm{kL}}{U_{0}}C^{*}$$

By introducing following dimensionless numbers, Péclet number, *Pe* and first Damköhler number, *Da*_I_, the equation can be finally transformed into:

(14) $$\frac{\partial C^{*}}{\partial t^{*}}+u^{*}\cdot \nabla C^{*}=\frac{1}{\mathrm{Pe}}\nabla^{2}C^{*}-Da_{I}C^{*}$$

where Péclet and first Damköhler numbers are described as follows:

(15) $$\mathrm{Pe}=\frac{U_{0}L}{D},Da_{I}=\frac{\mathrm{kL}}{U_{0}}$$

The *Pe* number indicates the ratio of convective mass transfer to molecular diffusion, and the *Da*_I_ number indicates the ratio of characteristic time for convection to characteristic time for chemical reaction. The focus in this study is the relationship between the macroscopic convective mass transfer and the chemical reaction rate. Therefore, we introduced *Da*_I_ to evaluate the effect of macroscopic mass transfer on the chemical reaction rate. The chemical reaction time is longer compared with the advection time and the concentration field becomes more uniform for *Da*_I_ ≪ 1. This condition is preferable to evaluate the whole chemical reaction rate, and the macroscopic mass transfer does not affect the whole reaction rate as shown in Fig. 1 (c). Meanwhile, the chemical reaction time is shorter compared with the advection time and the concentration field becomes non-uniform for *Da*_I_ ≫ 1. In this case, the whole chemical reaction rate is affected by the convective mass transfer as shown in Fig. 1 (a).

We calculated these dimensionless numbers based on the experimental results of chemical reaction rate as shown in Fig. 6 and of flow velocity as shown in Fig. 11. It is to be noted that the measured reaction rate constant was used to calculate these dimensionless numbers although the local reaction rate is not uniform spatially and the measured reaction rate constant was obtained for whole bath. That is, the reaction rate constant used in this calculation is underestimated. To calculate these dimensionless numbers, the maximum flow velocity obtained by the PIV measurement was used for the characteristic fluid velocity, and the depth of ultrasonic bath was used for the characteristic length. The calculated *Pe* and *Da*_I_ numbers for 50 W are 3.0 × 10^6^ and 1.9 × 10^-3^, respectively. The calculated *Pe* and *Da*_I_ numbers for 110 W are 3.0 × 10^6^ and 5.0 × 10^-3^, respectively. From this calculated result, it is obvious that the convective mass transfer is dominant compared with the diffusive mass transfer. Also, the effect of convective mass transfer on the chemical reaction rate is not large because the *Da*_I_ is smaller than 1. Furthermore, the spatial concentration variation in the ultrasonic bath is larger in the case of 110 W compared with 50 W as shown in Fig. 9, and this result is also reflected in the dimensionless number. This calculated dimensionless number corresponds to the experimental results obtained by the P-LIF experiment.

These results are very important to evaluate the effect of macroscopic convective mass transfer on the chemical reaction, and these results suggest that the *Da*_I_ number can be an indicator to evaluate the macroscopic mass transfer in an ultrasonic bath. We propose the following procedure to evaluate the effect of convective mass transfer on the sonochemical reaction rate.

1. Measure the reaction rate by UV–Vis absorption spectroscopy.
2. Calculate the rection rate constant, *k*
3. Measure the fluid flow velocity by the PIV measurement, *U*_0_
4. Calculate first Damköhler number, *Da*_I_

When the *Da*_I_ is much smaller than 1, the advection time is much shorter than the reaction time, resulting in more uniform concentration distribution in an ultrasonic bath. In this condition, the macroscopic mass transfer does not affect the whole chemical reaction rate. Meanwhile, the *Da*_I_ is close to 1 or larger than 1, the advection time is close or larger than the reaction time resulting in the larger spatial concentration variation. In this condition, the macroscopic mass transfer can affect the whole reaction rate.

In this equipment, the effect of macroscopic convective mass transfer on the reaction rate is small. However, this scenario can change in other equipment such as ultrasonic irradiation through a sonotrode [58,59], with gas control [56,57] and additional chemicals to enhance the chemical reaction rate such as CCl_4_, Fenton reagent, and etc. [[53], [54], [55]]. Under these conditions, the chemical reaction rate is enhanced, or the fluid velocity of acoustic streaming is changed. For example, the fluid velocity of acoustic streaming is very large and the reaction rate is small when ultrasound is irradiated by the sonotrode. In this case, the *Da*_I_ becomes small, estimating that the convective mass transfer less affects the chemical reaction rate. In this way, even under the different conditions with different equipment, frequency and dissolved gas concentration, the effect of mass transfer on the chemical reaction rate can be quantitatively evaluated by the *Da*_I_.

### 3.5 Comparison with other studies about mass transfer due to ultrasound

In this section, we compared the results in this study with those in the previous studies related to the mass transfer in an ultrasonic bath. Main interests related to the mass transfer are in an ultrasonic drying process and mass transfer enhancement across the gas–liquid or liquid–solid interface in many applications [[60], [61], [62]]. In these applications, the mass transfer is enhanced due to cavitation bubbles motion and capillary waves. However, in these studies, the focus is not on the relationship between the macroscopic mass transfer on the chemical reaction rate. There are a few studies which focus on the interaction between the macroscopic convective mass transfer and the chemical reaction [36,38,40,[63], [64], [65]]. As reviewed in introduction, the addition of external flow changes the ultrasonic and reaction fields [[37], [38], [39]]. Therefore, there are very few studies that isolate the effect of macroscopic mass transfer on the chemical reaction rate. Recently, numerical models to predict not only the ultrasound propagation but fluid flow and mass transfer have been developed. The effect can be quantitatively evaluated by the numerical simulation. Rahimi *et al*. [64] numerically modeled the mass transfer of oxidative desulfurization of fuel in an ultrasonic horn reactor. Komarov *et al*. [38], modeled the degradation of Rhodamine B in an ultrasonic circulatory reactor. In these studies, although the phenomena occurring in the reactors were numerically modeled, the experimental verification and validation were not discussed carefully. Also, the influence of mass transfer on the reaction rate was just explained by the turbulent diffusion. Recently, Goris *et al*. [65] investigated and modeled the transport phenomena and acoustic field simultaneously. This numerical model is quite useful and it was rigorously modeled. The chemical reaction and mass transfer were solved together. However, the relationship between the mass transfer and the chemical reaction rate was not discussed.

The macroscopic mass transfer is likely to be important in numerous ultrasonic applications. For example, the reaction rate of ultrasonic organic chemical decomposition and ultrasonic advanced oxidation process has been recently enhanced [[66], [67], [68]]. Under this condition, the macroscopic mass transfer easily affects the chemical reaction rate because the reaction rate constant becomes larger and the characteristic time of chemical reaction becomes shorter resulting in larger *Da*_I_ number. Therefore, the proposed method of P-LIF experiment and estimation method using *Da*_I_ number become important indicator. In future works, this relationship should be clarified in various conditions and with different equipment by using the *Da*_I_ number.

### 3.6 Limitation of this experimental method and estimation error to evaluate the effect of convective mass transfer on the chemical reaction rate

In this study, we first measured concentration field of Rhodamine in an ultrasonic bath through the P-LIF measurement. The limitation and experimental errors of this experiment are explained below. Generally, the P-LIF measurement is reliable method to quantify the concentration distribution in a cross-sectional plane, and this method has been widely used for various applications such as mixing [69,70] and mass transfer across the gas–liquid interface of bubble [71,72]. Readers can refer to the review paper for common errors arising from this measurement [73]. In addition to the common errors, the nucleated bubble on the vessel walls becomes main factors to cause large measurement error in an ultrasonic bath. As mentioned in the results, the bubbles on the walls scatter and attenuate the laser light causing fluorescence intensity change recorded by the camera. Therefore, this method is not preferable for long time observation in an ultrasonic bath. In addition, the temperature variation can change the fluorescence intensity. Even with Rhodamine 6G, the sensitivity of fluorescence for temperature is 0.003 /K [42]. The temperature increase with 110 W power was approximately 3 K for 120 s irradiation and the error caused by temperature variation was less than 1 %. Therefore, the fluorescence change due to temperature variation was negligibly small in this study. However, one should remind that this method cannot be used for an ultrasonic bath with large temperature variation. In addition, the sensitivity of Rhodamine B is approximately 5 times larger than that of Rhodamine 6G. Therefore, the tracer should be carefully selected. Photobleaching is another problem as explained in the experimental procedure. The laser intensity should be adjusted for fluorescent chemicals not to be photobleached.

The P-LIF method has some advantages compared with other measurement methods such as KI dosimetry, decomposition method, SCL observation and etc. In this method, the concentration distribution of solute can be directly measured quantitatively. Therefore, this measurement can help to construct a way to improve convective mass transfer in an ultrasonic bath. On the contrary, the reaction rate cannot be accurately measured by this measurement. Therefore, the combination of the P-LIF method and the proposed procedures using *Da*_I_ number is the most preferable method when improving macroscopic mass transfer.

Next, we discuss the estimation error to evaluate the effect of convective mass transfer on the chemical reaction rate by means of *Da*_I_. The *Da*_I_ is calculated by the experimentally measured fluid flow velocity and reaction rate constant. This reaction rate constant is different from the actual rate constant because the reaction zone is limited and the influence of mass transfer on the reaction rate is also included. Therefore, the calculation error becomes large when the reaction zone is limited in a narrow zone. In addition, the flow velocity is overestimated because the maximum velocity is used instead of averaged flow velocity in a direction. Therefore, it is not always the case that the phenomenon is changed when the *Da*_I_ is 1. The evaluation method proposed in this study should be used with consideration of these errors.

## 4 Conclusion

In the present study, we investigated the influence of macroscopic mass transfer on the reaction rate through the P-LIF measurement for an ultrasonic degradation to answer to the question whether the macroscopic mass transfer affects the sonochemical reaction rate in an ultrasonic bath or not. The concentration distribution of Rhodamine 6G is measured by the P-LIF measurement and we discussed the obtained concentration distribution using the measured distribution and rate of chemical reaction, and the measured distribution of flow velocity. The following conclusions and achievement can be made from the results:

• The macroscopic concentration distribution in an ultrasonic vessel can be measured for the first time by the P-LIF measurement although the measurement errors caused by the bubbles at the vessel wall remain.
• The concentration of Rhodamine 6G decreases first in the zone with high reaction rate, and then the chemical species are transported to the low-reaction zone by the macroscopic convective mass transfer through solute plumes.
• In our experimental apparatus, the concentration is spatially varied by approximately 20 % at most in the early stage of ultrasonic degradation because the reaction rate is highest in this stage.
• The apparent reaction rate slightly differs from the actual reaction rate in the early stage of ultrasonic degradation. The adequate sampling interval of dosimetry can avoid this error under the condition where the influence of macroscopic convective mass transfer on the chemical reaction rate is large.
• The first Damköhler number, *Da*_I_ can quantitatively evaluate the influence of macroscopic mass transfer on the chemical reaction rate. This number can be calculated by conducting the measurements of reaction rate constant and fluid flow velocity.

## CRediT authorship contribution statement

**Takuya Yamamoto:** Writing – review & editing, Writing – original draft, Visualization, Software, Methodology, Investigation, Funding acquisition, Formal analysis, Data curation, Conceptualization. **Shinya Okino:** Writing – review & editing, Methodology.

## Declaration of competing interest

The authors declare the following financial interests/personal relationships which may be considered as potential competing interests: Takuya Yamamoto reports financial support was provided by Japan Science and Technology Agency. If there are other authors, they declare that they have no known competing financial interests or personal relationships that could have appeared to influence the work reported in this paper.

## Figures and Tables

**Fig. A1:**
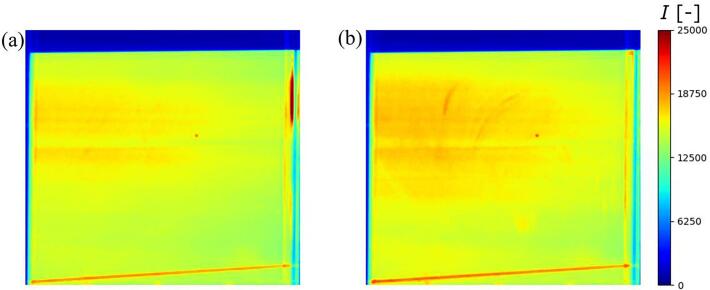
The distribution of fluorescence light intensity at (a) 0 and (b) 1200 s. The concentration of Rh6G is 10 μg/L.

**Fig. A2:**
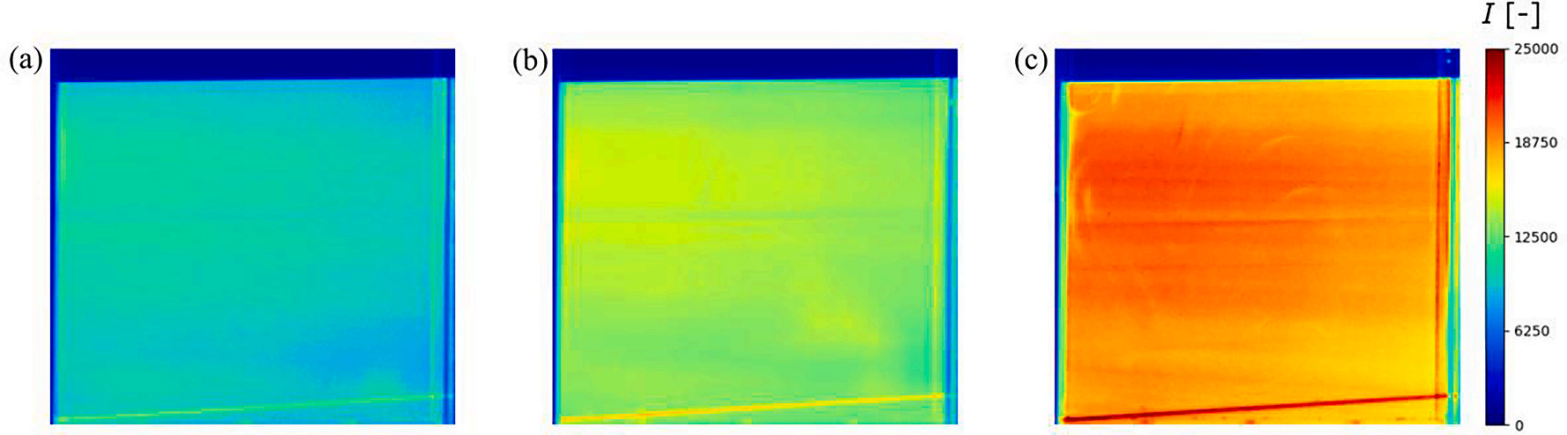
Reference fluorescence intensity with different solution concentration of Rh6B: (a) 5.0 μg/L, (b) 7.5 μg/L and (c) 10 μg/L.

## Appendix

### Appendix A Photobleaching of Rhodamine 6G due to laser irradiation

The fluorescence can be degraded by a laser irradiation, and this phenomenon is called photobleaching [43,73,74]. To evaluate this photobleaching on the results, we continued to irradiate the laser sheet to the Rh6G aqueous solution without ultrasonic irradiation. Fig. A1 shows the distribution of fluorescence light intensity at 0 and 1200 s. The fluorescence intensity distribution is not changed very much or slightly increased because the laser output is slightly varied temporary. In this experiment, the standard deviation of laser output is 0.261 %, and this difference is in the range of this laser output variation. Hence, the degradation of fluorescence intensity due to laser irradiation is negligibly small.

## Appendix B Reference fluorescent intensity distribution used in the P-LIF experiment

Fig. A2 shows the reference fluorescence intensity with different solution concentration of Rh6B. The fluorescence intensity is absent at the top of each picture because the laser sheet is shaded near the free surface to prevent irregular reflection of the laser sheet. Also, the fluorescence intensity is low at the right and left sides due to the acrylic vessel wall. The laser sheet is also reflected at the bottom surface, and the reflected light creates the high-intense striped zone near the bottom surface. The fluorescence intensity decreases monotonically in accordance with the solution concentration. By using this reference data, the solution concentration was finally calculated.

## Appendix C Interpretation for the definition of first Damköhler number

In this study, we introduced the first Damköhler number as *Da*_I_ = *kL*/*U*_0_. Generally, first Damköhler number is defined as

(A1) $$Da_{I}=\frac{t_{f}}{t_{c}}$$

where *t*_f_ is the characteristic time for flow and *t*_c_ is the characteristic time for chemical reaction. We adopted the time taken for fluid to reach the free surface from the vessel bottom wall as the characteristic time for flow, *t*_f_.

(A2) $$t_{f}=L/U_{0}$$

The maximum flow velocity was used as the characteristic fluid velocity, *U*_0_, In addition, the characteristic time for chemical reaction, *t*_c_ was derived from the non-dimensionalized equation as in Eq. (15).

(A3) $$t_{c}=1/k$$

This characteristic time for chemical reaction was interpretated from Eq. (3). By transforming Eq. (3), the following equation was obtained.

(A4) $$t=\frac{1}{k}\ln\left(\frac{C_{0}}{C}\right)$$

From Eqs. (A3), (A4), we can consider the ln(*C*_0_/*C*) to be 1. That is, the characteristic time for reaction is reaction time when the concentration in the solution becomes lower than *C*_0_/*e*, where *e* is Napier’s constant.
